# Chiral Organic Chromophoric Systems in the Enhancement of Circularly Polarized Luminescence

**DOI:** 10.3389/fchem.2021.635655

**Published:** 2021-02-23

**Authors:** Tao Wu, You-Xuan Zheng, Giovanna Longhi, Ga-Lai Law

**Affiliations:** ^1^ Institute of Organic Chemistry and Biochemistry, Academy of Sciences of the Czech Republic (ASCR), Prague, Czechia; ^2^ Nanjing University, Nanjing, China; ^3^ University of Brescia, Brescia, Italy; ^4^ Hong Kong Polytechnic University, Kowloon, Hong Kong

**Keywords:** circularly polarized luminescence, organic chromophore, molecular chirality, excitated state, chiral enhancement

Circularly polarized luminescence (CPL) discloses rich information about molecular chirality in the excited state. The number of reports on CPL studies has increased significantly since 2010: with the progress of instrumental techniques and calculation methods, CPL is widely used in the development of smart materials for advanced photonic technologies, 3D display and bio-responsive studies.

Recently, several significant overviews have been presented on CPL instrumental development and theoretical interpretation (Longhi et al., 2016), CPL of lanthanide complexes (Zinna and Di Bari, 2015), small organic molecules (Sanchez-Carnerero et al., 2015; Mori, 2020) and supramolecular assemblies (Kumar et al., 2015). A key factor to evaluate the enhancement of CPL is the dissymmetry factor *g*_lum_. A general theoretical aspect to achieve high value of the *g*_lum_ is the manipulation of optical transitions with strong magnetic and weak electric dipole transition moment contribution.

This Special Issue includes 12 contributions (1 Perspective, 2 Reviews, 3 mini-reviews, and 6 research papers) that touch experimental and theoretical aspects of CPL enhancement studies, including small organic molecules, polymers, aggregates, and metal complexes.

In a perspective paper, Kondo et al. summarize the KBr pellet method for CPL measurement, providing a protocol for solid-state CPL measurement and temperature-dependent samples.

Doistau et al. discuss the strategies to obtain strong CPL activity, and compare optical properties of CPL materials based on chiral d-block and f-block metal complexes, demonstrating an empirical protocol for CPL applications with cheap transitional metals.

Gao et al. review the most recent advances in switchable CPL aspects toward external stimuli, giving an overview of CPL switches for various technical applications.

Nagata and Mori present a specific mini-review on simultaneous improvement of luminescence quantum yields and dissymmetry factors, paving a way for obtaining excellent organic CPL materials.

A special focus on cyclophane-based chromophore in CPL materials is reported in the mini-review by Sugiura. Upon synthetic chemical tools, one may manipulate the orientations of fluorophores and obtain strong CPL materials as desired.

In the mini-review by Kumar et al., they discuss several chiral templates such as liquid crystals, biomolecules, molecular self-assemblies and chiral gelators utilized for generating enhanced CPL in organic luminophores.

Theoretical evaluations on electric dipole forbidden and magnetic dipole allowed transitions represent a particularly appealing approach for understanding the origin of CPL, thus aiding the interpretation of CPL enhancement mechanism.

Del Galdo et al. introduce an economic computational way for the simulation of solvatochromic shifts of medium-size flexible chromophores in condensed phases. Yang et al. present an extensive protocol for the interpretation of CPL spectra of medium-to-large molecular systems. Incorporation of vibrational participations provides comprehensive insights on the origin of the measured spectral band-shape.

From an industrial point of view, CPL enhancement plays a key role in the development of chiral materials applied in optoelectronic devices, e.g. circularly polarized organic light-emitting diodes (CP-OLEDs), chiral photovoltaics and transistors.

Dhbaibi et al. report on the synthesis of chiral diketopyrrolopyrrole-helicene polymers showing red CPL, which gives a fascinating example demonstrating the potential of π-conjugated helical polymers for chiral optoelectronic applications.

In another research paper, Reine et al. present an enantiopure bis-perylenediimide cyclohexane derivative with CPL response. The optical properties can be easily tuned by self-assembly or by functionalization of the electron-deficient organic chromophore.

The diversity of coordination environment with chiral ligands makes Pt(II) complexes quite appealing for CPL studies. Yang et al. present a case on manipulation of CPL enhancement in phosphorescent chiral Pt(II) complexes by inclusion of bulky ligands.

Finally, Yan et al. present the design and synthesis of a pair of chiral platinahelicene enantiomers with CPL activity. The evaporated CP-OLEDs based on platinahelicene display deep-red emission as well as distinct CP electroluminescence (CPEL) signals, providing an excellent example to improve the CPL properties of organic chromophore to cope with the future application in CP-OLEDs.

The editorial team would like to thank all authors for their excellent contributions to this exciting topic. The Editors suggest that such a rich palette of articles, manifests the essential features of the chiral chromophores in the enhancement of CPL, and will serve to guide and promote future development in the field.
